# *Echinococcus granulosus* Antigen B Structure: Subunit Composition and Oligomeric States

**DOI:** 10.1371/journal.pntd.0001551

**Published:** 2012-03-06

**Authors:** Karina M. Monteiro, Mateus B. Cardoso, Cristian Follmer, Nádya P. da Silveira, Daiani M. Vargas, Elliot W. Kitajima, Arnaldo Zaha, Henrique B. Ferreira

**Affiliations:** 1 Laboratório de Biologia Molecular de Cestódeos and Laboratório de Genômica Estrutural e Funcional, Centro de Biotecnologia, Universidade Federal do Rio Grande do Sul, Porto Alegre, Rio Grande do Sul, Brazil; 2 Laboratório Nacional de Luz Síncrotron (LNLS), Campinas, São Paulo, Brazil; 3 Departamento de Físico-Química, Instituto de Química, Universidade Federal do Rio de Janeiro, Rio de Janeiro, Rio de Janeiro, Brazil; 4 Instituto de Química, Universidade Federal do Rio Grande do Sul, Porto Alegre, Rio Grande do Sul, Brazil; 5 Departamento de Entomologia, Fitopatologia e Zoologia Agrícola, Escola Superior de Agricultura Luiz de Queiroz (ESALQ), Universidade de São Paulo, Piracicaba, São Paulo, Brazil; Asahikawa Medical College, Japan

## Abstract

**Background:**

Antigen B (AgB) is the major protein secreted by the *Echinococcus granulosus* metacestode and is involved in key host-parasite interactions during infection. The full comprehension of AgB functions depends on the elucidation of several structural aspects that remain unknown, such as its subunit composition and oligomeric states.

**Methodology/Principal Findings:**

The subunit composition of *E. granulosus* AgB oligomers from individual bovine and human cysts was assessed by mass spectrometry associated with electrophoretic analysis. AgB8/1, AgB8/2, AgB8/3 and AgB8/4 subunits were identified in all samples analyzed, and an AgB8/2 variant (AgB8/2v8) was found in one bovine sample. The exponentially modified protein abundance index (emPAI) was used to estimate the relative abundance of the AgB subunits, revealing that AgB8/1 subunit was relatively overrepresented in all samples. The abundance of AgB8/3 subunit varied between bovine and human cysts. The oligomeric states formed by *E. granulosus* AgB and recombinant subunits available, rAgB8/1, rAgB8/2 and rAgB8/3, were characterized by native PAGE, light scattering and microscopy. Recombinant subunits showed markedly distinct oligomerization behaviors, forming oligomers with a maximum size relation of rAgB8/3>rAgB8/2>rAgB8/1. Moreover, the oligomeric states formed by rAgB8/3 subunit were more similar to those observed for AgB purified from hydatid fluid. Pressure-induced dissociation experiments demonstrated that the molecular assemblies formed by the more aggregative subunits, rAgB8/2 and rAgB8/3, also display higher structural stability.

**Conclusions/Significance:**

For the first time, AgB subunit composition was analyzed in samples from single hydatid cysts, revealing qualitative and quantitative differences between samples. We showed that AgB oligomers are formed by different subunits, which have distinct abundances and oligomerization properties. Overall, our findings have significantly contributed to increase the current knowledge on AgB expression and structure, highlighting issues that may help to understand the parasite adaptive response during chronic infection.

## Author Summary

Antigen B (AgB) is the major secretory protein of the *Echinococcus granulosus* hydatid cyst, the causative agent of cystic hydatid disease. Structurally, AgB is a multisubunit protein formed by 8-kDa subunits, but it is not known which subunits are secreted by a single parasite (cyst) and how they interact in the formation of distinct AgB oligomeric states. Here, we investigated AgB subunit composition and oligomeric states in individual samples from bovine and human cysts. We identified AgB8/1, AgB8/2, AgB8/3 and AgB8/4 subunits in AgB oligomers of all samples analyzed. Quantitative and qualitative differences in the expression of AgB subunits were observed within and between samples. Using recombinant subunits as models, we showed that AgB subunits form distinct oligomeric states, with a rAgB8/3>rAgB8/2>rAgB8/1 maximum size relation. We also demonstrated by different experimental approaches that rAgB8/3 oligomers are more similar, both in size and morphology, to those observed for *E. granulosus* AgB. Overall, we provided experimental evidences that AgB is composed of different subunits within a single cyst, and that subunits have different abundances and oligomerization properties. These issues are important for the understanding of AgB expression and structure variations, and their impact for the host-parasite cross-talk.

## Introduction

*Echinococcus granulosus* is the causative agent of cystic hydatid disease (CHD), a worldwide zoonotic infection that affects humans and livestock [1]. Antigen B (AgB) is the major protein secreted by the *E. granulosus* pathogenic larval stage (metacestode or hydatid cyst). Since its first description in 1971 [2], AgB has been the most studied *E. granulosus* protein due to its role in parasite biology and its potential for application in CHD control tools [3], [4]. AgB has been described as involved in several host-parasite interaction mechanisms that promote parasite establishment and survival in the intermediate host, such as protease inhibition [5], lipid binding [6] and immunomodulation [7], [8]. Furthermore, AgB is highly immunogenic in human infections, presenting a high diagnostic value for CHD [9], [10].

AgB is homologous to hydrophobic ligand binding proteins (HLBPs), a family of cestode helix-rich proteins that bind hydrophobic compounds [6]. It is an oligomeric lipoprotein composed of 8-kDa related subunits (AgB8 subunits) [2], [11], which are encoded by a multigene family that includes at least five members (*AgB1-5*) [12]–[16]. In SDS-PAGE, under reducing conditions, AgB dissociates into low-order oligomers of 8-kDa subunits (monomers, dimers, trimers, etc.) [11], [17]. In solution, AgB exists as high-order oligomers [2], [18], showing distinct populations of ∼160, ∼240 and >320 kDa.

Despite being an extensively studied protein, several important aspects of the AgB molecular structure remain largely uncharacterized [19], such as its subunit composition and oligomeric states. It is not known which AgB subunits are expressed and secreted in the hydatid fluid of an individual cyst, as studies carried out so far analyzed AgB from a pool of cysts [17], [20]. The abundance of each 8-kDa subunit within a cyst, its oligomerization properties, and its contribution to define the distinct AgB oligomeric states are also still open questions.

AgB subunits could present differential expression patterns within individuals [21] and/or throughout the parasite's development [22], [23]. Furthermore, different 8-kDa subunits have distinct physical-chemical [18], immunological [9] and lipid-binding [6] properties. So, AgB subunit composition and abundance may determine distinct protein oligomeric states, biochemical interactions and biological roles. Therefore, these AgB structural aspects are expected to have repercussions on adaptive host-parasite relationships and on the outcome of AgB-based CHD immunodiagnostic methods [19], [21]. Thus, there is a clear need for further studies to elucidate which subunits are secreted in the hydatid fluid of a cyst and to characterize the oligomeric states formed by these subunits. This information could aid in a better understanding of the roles played by AgB during the host-parasite interaction and improve its application in advancing immunodiagnostic and therapeutic approaches for CHD.

Recently, we demonstrated the self-assembly of three AgB recombinant subunits (rAgB8/1, rAgB8/2 and rAgB8/3) into homo-oligomers that have similar properties to those of parasite-produced AgB, validating them as tools for studying AgB structure [18]. In the present work, we investigated the subunit composition and oligomeric states of *E. granulosus* AgB. The subunit composition of AgB purified from individual bovine and human hydatid cysts was studied by mass spectrometry associated with electrophoretic analysis. The exponentially modified protein abundance index (emPAI) was employed to obtain information on the relative abundance of the 8-kDa subunits within the different AgB samples. Using the available AgB recombinant subunits, we assessed the *in vitro* oligomerization properties of these different 8-kDa subunits and performed a comparative structural characterization of the recombinant oligomers and AgB purified from hydatid cyst.

## Methods

### Parasite material and AgB purification

*E. granulosus* bovine hydatid cysts were obtained from lungs of naturally infected animals slaughtered at Frigorífico Cooperleo, São Leopoldo, RS, Brazil. Animal slaughtering was conducted according to Brazilian laws and under supervision of the *Serviço de Inspeção Federal* (Brazilian Sanitary Authority) of the Brazilian *Ministério da Agricultura*, *Pecuária e Abastecimento*. Condemned viscera at post-mortem meat inspection due to the presence of hydatid cysts were collected at the abattoir and dissected in the laboratory, following protocols approved by the Ethical Committee of the *Universidade Federal do Rio Grande do Sul*. For AgB purification from single cysts, hydatid fluid samples from two fertile cysts (collected from different animals) were aseptically aspirated and individually processed for immunopurification. Hydatid fluid samples were processed according to Oriol et al. [2] and AgB immunopurification was performed as described previously [18]. Cysts used in this work were required to have a minimum volume of ∼200 ml hydatid fluid, which was necessary to obtain enough AgB for mass spectrometry and structural analysis. Maximum yields of 1 mg AgB were typically obtained from each bovine cyst.

Human hydatid fluid was collected from a CHD patient after surgical aspiration of a lung cyst content performed at Instituto Hugolino Andrade, Santana do Livramento, RS, Brazil. The cyst was fertile and classified as type CE1 according to the standardized WHO classification [24]. The patient did not undergo any chemotherapy prior to surgery, and signed an informed consent for both the surgical procedure and the use of the aspired hydatid fluid for research purposes. The use of this biological sample was approved by the Ethical Committee of the *Universidade Federal do Rio Grande do Sul*. The hydatid fluid was clarified by centrifugation at 10000× g for 15 min at 4°C and concentrated 10-fold by vacuum centrifugation prior to electrophoretic analyses. AgB was not purified and the analyses were performed with raw human hydatid fluid, as it showed large quantities of AgB and minimal contamination with host proteins.

Bovine and human cysts used in this work were genotyped as *E. granulosus* sensu stricto G1 (sheep strain) (for details, see Text S1).

### AgB recombinant subunits

AgB recombinant subunits rAgB8/1, rAgB8/2 and rAgB8/3 were expressed in *Escherichia coli* as glutathione S-transferase fusion proteins, purified by affinity chromatography and recovered using thrombin cleavage as described previously [25]. Protein concentrations were determined using a Qubit quantitation fluorometer and Quant-it reagents (Invitrogen, Carlsbad, USA).

### Polyacrylamide gel electrophoresis (PAGE)

For SDS-PAGE analyses, AgB samples (40 µg) were resolved on 15% gels, under reducing conditions, as described by Laemmli [26]. Native PAGE was performed in 4–20% Mini-PROTEAN TGX gels (Bio-Rad, Hercules, USA) using Tris-glycine (pH 8.3) as running buffer. Protein samples (10 µg) were diluted with an equal volume of 2× native sample buffer (62.5 mM Tris-HCl, pH 6.8, 40% glycerol, 0.01% bromophenol blue) and run at a constant voltage of 100 V. Both denaturing and native gels were stained with Coomassie blue and scanned with an ImageScanner (GE Healthcare, Chalfont St. Giles, UK).

### Mass spectrometry (MS) analysis

#### In-solution and in-gel digestion

Protein samples (100–200 µg) were digested in solution with trypsin as described previously [27], and the resulting peptides were desalted using OASIS HLB Cartridge (Waters, Milford, USA). For in-gel digestion, AgB bands resolved by SDS-PAGE or native PAGE were excised from the gels and destained by three washes with 25 mM NH_4_HCO_3_/50% acetonitrile and then dehydrated by addition of 100% acetonitrile for 5 min. The liquid phase was removed, and the gel pieces were completely dried in a vacuum centrifuge. Dried gels were treated with 10 mM dithiothreitol (DTT) in 25 mM NH_4_HCO_3_ and proteins were reduced for 1 h at 56°C. The DTT solution was then replaced by 55 mM iodoacetamide in 25 mM NH_4_HCO_3_. After 45 min at room temperature in the dark, the gel pieces were washed twice with 25 mM NH_4_HCO_3_ and dehydrated by addition of 100% acetonitrile. After completely dried in a vacuum centrifuge, the gel pieces were rehydrated with trypsin solution (10 µg/ml trypsin in 25 mM NH_4_HCO_3_). The enzymatic reaction was allowed to proceed for 16–20 h at 37°C. Peptides were extracted twice by incubation with 50% acetonitrile/5% trifluoroacetic acid (TFA) for 1 h. The extractions were combined and dried in a vacuum centrifuge. Peptides were resuspended in 12 µl of 0.1% TFA and 5 µl were analyzed by MS.

#### MS analysis and data processing

The peptides were analyzed by on-line liquid chromatography/mass spectrometry (LC-MS/MS) using a Waters nanoACQUITY UPLC system coupled to a Waters Micromass Q-Tof Micro or Q-Tof Premier mass spectrometer (Waters MS Technologies, Manchester, UK). The peptides were eluted from the reverse-phase column towards the mass spectrometer at a flow rate of 200 nl/min, with a 10–50% water/acetonitrile 0.1% formic acid linear gradient over 30 min. Analyses were performed using the data-dependent acquisition (DDA) mode. Automatic switching from MS to MS/MS was performed for precursor ion counts that were greater than 8, and the MS/MS collision energy was dependent on the precursor ion m/z and charge state. MS experiments were performed in duplicates.

MS/MS raw data were processed using Mascot Distiller 2.2.1 (Matrix Science, London, UK), and the peak lists were exported in Mascot Generic Format (.mgf). Searches were performed in a local *E. granulosus* protein database and in public databases. Database searches were performed using MASCOT software (Matrix Science, London, UK) with the following search parameters: a maximum of one missed cleavage site, fixed carbamidomethyl alkylation of cysteines, variable oxidation of methionines and a 0.1 mass unit tolerance on parent and fragment ions. The significance threshold was set at p<0.05, and only peptides with individual ion scores above this significance threshold (typically >14 for the *E. granulosus* database) were considered for protein identification.

The exponentially modified protein abundance index (emPAI) was used to estimate the relative abundance of 8-kDa subunits in each AgB sample. MASCOT software was used to calculate emPAI values by normalizing the number of experimentally observed peptides per protein by the theoretical number of peptides per protein [28]. The mean emPAI values obtained from the two MS replicates of each sample were used for protein abundance analysis.

### Light scattering (LS) measurements

LS measurements of AgB samples purified from bovine cysts were performed on a DynaPro instrument (Protein Solutions Inc., Charlottesville, USA), as it allows for the use of smaller sample volumes, compatible with the low yields obtained from *E. granulosus* AgB purification. AgB was analyzed in PBS (phosphate buffered saline, pH 7.4) at a concentration of 100 µg/ml, and DynaPro V.5 software was used for data analysis.

AgB recombinant subunits were analyzed on a Brookhaven Instruments standard setup (BI-200 M goniometer, BI-9000 AT digital correlator) with a He-Ne laser (λ = 632.8 nm) as light source. Proteins samples were diluted in PBS to a final concentration of 1 mg/ml. After centrifugation (14,000 rpm, 10 min, 4°C), the supernatants were filtered through 0.22 µm pore diameter membranes (Millipore, Milford, MA, USA) into dust-free cylindrical cuvettes in a laminar flow box. Measurements of both dynamic and static light scattering (DLS and SLS, respectively) were conducted in triplicate at a scattering angle of 90°. A water bath was used to control the temperature and LS was measured at 23°C and 37°C. The samples were equilibrated at each temperature for 10 min before the LS measurements. DLS and SLS data analysis was performed as described in Text S1.

### Microscopy

#### Sample preparation

*E. granulosus* AgB and recombinant oligomers were analyzed by microscopy at concentrations of 3 mg/ml in PBS. In order to characterize the oligomeric states formed by recombinant subunits at physiological conditions, AgB subunits were incubated at 37°C for 15 min prior to processing for microscopy analyses.

#### Transmission electron microscopy (TEM)

Formvar/carbon-coated grids were placed over 15 µl drops from each protein sample. After 15 min, the excess sample was removed using filter paper, and the grids were washed three times with distilled water. The bound particles were stained with 30 µl uranyl acetate (1% aqueous solution) for 5 min and examined in a Zeiss EM 900 electron microscope operating at 50 kV.

#### Atomic force microscopy (AFM)

Three microliters of each protein sample were deposited onto silicon substrates previously cleaned with ethanol and acetone. After 60 s, the excess sample was removed by wicking with filter paper, and the substrates were gently rinsed twice with 50 µl of MilliQ water to remove salts and loosely bound proteins. The samples were dried under normal atmospheric conditions at room temperature and then imaged with a DI Nanoscope IIIa microscope in the noncontact mode. The images were obtained in the air under ambient conditions at a scan rate of 0.8 Hz. AFM images were analyzed using WSxM v4.0 software (Nanotec) [29].

### Pressure-induced dissociation of AgB recombinant oligomers

The recombinant AgB oligomers (0.2 mg/ml in 25 mM Tris, pH 7.5) were subjected to high hydrostatic pressure treatment at 25°C in either the absence or presence of 2-mercaptoethanol. The high pressure cell, equipped with optical windows, has been previously described [30]; it was purchased from ISS (Champaign, IL). The average size distribution of the proteins in solution was evaluated by exciting the samples at 320 nm and collecting the scattered light at 90° from 315 to 325 nm in an ISS K2 spectrofluorometer. For a compression/decompression cycle, the pressure was increased from 1 to 3000 bar in steps of 270 bar and then decreased in similar steps. At each step, the samples were allowed to equilibrate for 15–20 min before LS data collection. The secondary structure content of proteins before and immediately after the compression was monitored by circular dichroism (CD).

## Results

### AgB subunit composition

The qualitative and quantitative subunit composition of AgB from individual bovine and human hydatid cysts was analyzed and compared using mass spectrometry. Total AgB subunit composition was analyzed by in-solution digestion of samples, while the specific composition of AgB low- and high-order oligomers was investigated by in-gel digestion of samples resolved in SDS-PAGE and native PAGE, respectively.

**Figure 1:**
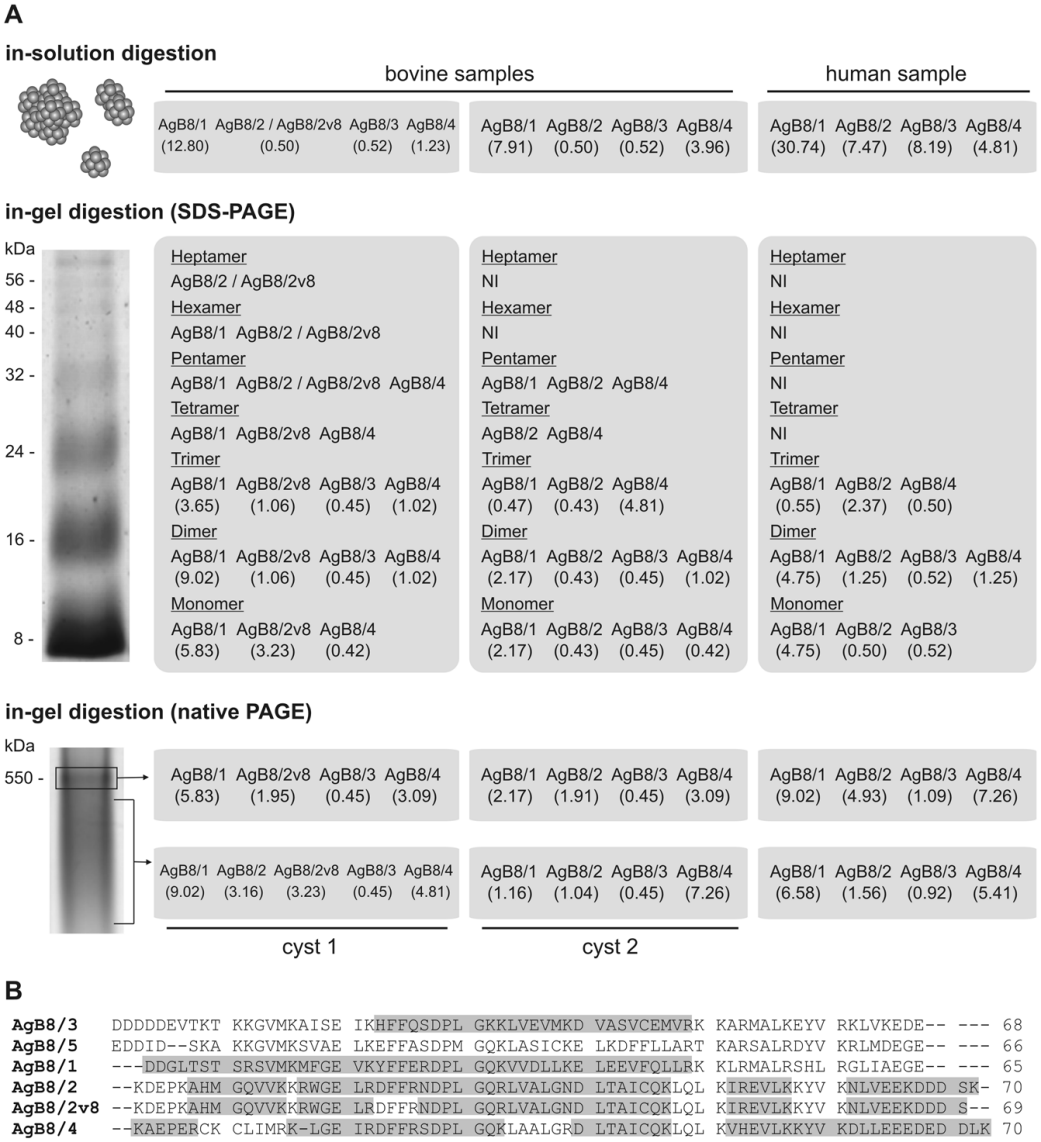
Qualitative and quantitative AgB subunit composition from bovine and human cysts. (A) Diagrammatic summary of the AgB subunits identified by mass spectrometry from in-solution and in-gel digested bovine and human samples. The calculated emPAI values are shown in parentheses. AgB8/2/AgB8/2v8 indicates that the protein isoforms can not be distinguished from the set of identified peptides. NI, no identification. (B) Alignment of the AgB mature peptides showing the sequences identified by mass spectrometry highlighted in gray. Sequence accession numbers: AgB8/1 (AAD38373), AgB8/2 (AAC47169), AgB8/2v8 (AAS88244), AgB8/3 (AAK64236), AgB8/4 (AAQ74958), and AgB8/5 (BAE94835).

Peptides corresponding to the AgB8/1, AgB8/2, AgB8/3, and AgB8/4 subunits were identified in all samples analyzed (Figure 1A and Table S1), while AgB8/5 subunit was not detected in any of them. Qualitative differences were observed between samples, with the identification of a variant for AgB8/2 subunit in one of the bovine samples (cyst 1). The detected AgB8/2 isoforms differ at their stop codon position, being the identification of the AgB8/2v8 variant based on the detection of the tryptic C-terminal peptide NLVEEKDDDS (Figure 1B). Mass spectrometry data from native PAGE revealed that the two AgB8/2 isoforms were expressed in the bovine cyst 1.

To investigate the relative abundance of different subunits within AgB samples we used the emPAI (Figure 1A and Table S1), which revealed a consistent and significant relative overrepresentation of AgB8/1 subunit in all analyzed samples. AgB8/2 and AgB8/4 subunits were also well represented in bovine and human samples, especially in high-order AgB oligomers resolved by native PAGE. On the other hand, AgB8/3 subunit relative abundance varied between AgB samples from different hosts. In bovine samples, AgB8/3 was the less abundant subunit, whereas in the human sample it was found in relative high abundance based on in-solution digestion data.

### AgB oligomeric states

**Figure 2:**
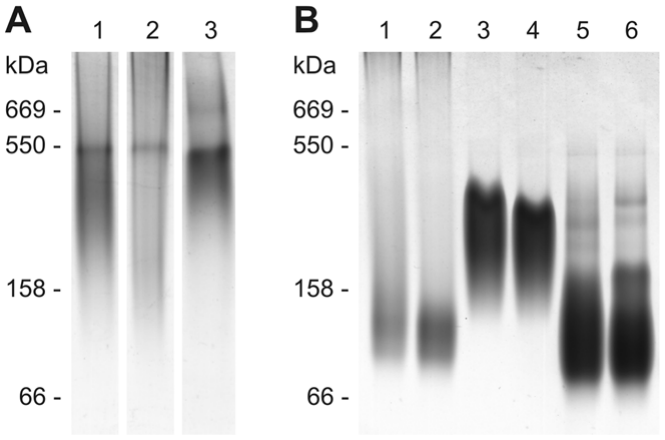
Native PAGE of *E. granulosus* AgB and recombinant oligomers. (A) AgB oligomers from bovine and human samples. Lanes 1–2, AgB purified from bovine cyst 1 and 2, respectively; lane 3, hydatid fluid from human cyst. (B) Oligomers formed by AgB recombinant subunits at different temperatures. Lanes 1–2, rAgB8/1; Lanes 3–4, rAgB8/2; Lanes 5–6, rAgB8/3, at 23°C (lanes 1, 3 and 5) and 37°C (lanes 2, 4 and 6). Samples were resolved on 4–20% polyacrylamide gels followed by Coomassie blue staining. The migration of molecular mass markers is indicated on the left of each gel. Protein markers were bovine thyroglobulin (669 kDa), *Helicobacter pylori* urease (550 kDa), bovine gamma-globulin (158 kDa) and bovine albumin (66 kDa).

AgB was found in different oligomeric states, as detected by both native PAGE and light scattering. In native PAGE, AgB showed oligomers of different sizes, which appeared as a broad smear, with a more defined band of ∼550 kDa (Figure 2A). In DLS, AgB also showed different oligomeric states, which are represented by populations with hydrodynamic radii (*R_h_*) of ∼4 nm, ∼100–200 nm and >2 µm. AgB samples heated at 37°C were also analyzed to evaluate the protein oligomeric states at physiological conditions, but no effect of temperature in AgB oligomerization was observed by native PAGE or DLS (data not shown).

In addition to AgB identification, the MS analysis of native PAGE bands from the human hydatid fluid revealed that the ∼550 kDa band also contained peptides corresponding to antigen 5 (Ag5), another *E. granulosus* secreted protein. Moreover, the ∼660 kDa band observed in this sample was also identified by MS as Ag5. These results (data not shown) are suggestive that Ag5 is also able to form high-order oligomers, as AgB.

In order to assess the oligomerization behavior of different AgB subunits, the homo-oligomers formed by the available recombinant subunits, rAgB8/1, rAgB8/2 and rAgB8/3, were also analyzed by native PAGE and light scattering. Both techniques detected a differential oligomerization behavior for AgB recombinant subunits, which formed oligomers with a maximum size relation of rAgB8/3>rAgB8/2>rAgB8/1. In native PAGE, the recombinant oligomers exhibited different size ranges, with rAgB8/3 forming the most heterogeneous oligomeric states, distributed over a wide range (∼100–550 kDa) (Figure 2B). This subunit also showed the most significant changes at 37°C, clearly exhibiting a higher oligomerization in response to temperature increasing. In addition, rAgB8/3 was the only subunit able to form higher-order oligomers (∼550 kDa) as those observed for *E. granulosus* AgB.

**Figure 3:**
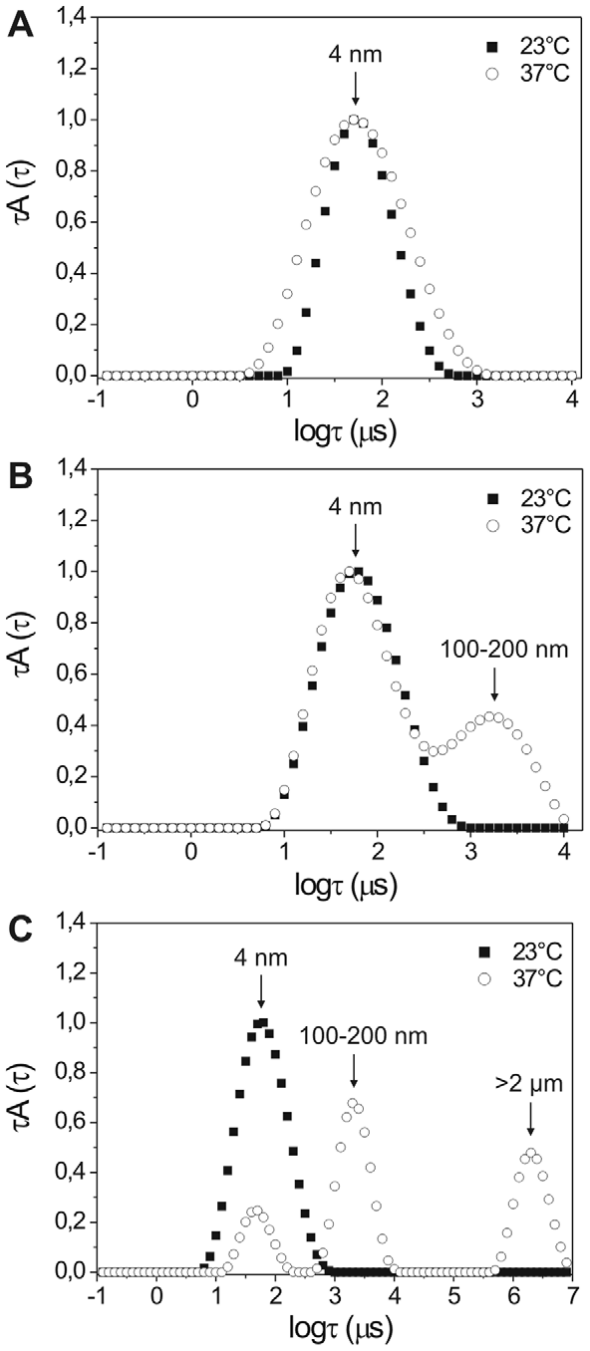
Oligomeric states formed by AgB recombinant subunits monitored by DLS as a function of temperature. Relaxation time distributions for rAgB8/1 (A), rAgB8/2 (B), and rAgB8/3 (C) at 23°C and 37°C. The relaxation time distributions are presented here as τA(τ) versus log τ (µs), where τA(τ) is the relaxation time, τ, multiplied by the scattering amplitude A(τ). The calculated hydrodynamic radii corresponding to each peak are indicated. DLS was recorded at a scattering angle of 90° from 1 mg/ml protein samples in PBS (pH 7.4).

The oligomeric states detected for rAgB8/3 subunit by DLS at physiological conditions, with calculated *R_h_* of ∼4 nm, ∼100–200 nm and >2 µm, were also more similar to those observed for *E. granulosus* AgB by the same technique. The oligomers of different sizes formed by each recombinant subunit at 37°C can be observed from relaxation time distributions in Figure 3. The relaxation time (τ) is inversely proportional to the diffusion coefficient of the scattering molecules, and hence directly proportional to their size. Thus, a fast relaxation time (in µs) was related to the motion of smaller particles and a slow relaxation time was related to that of larger particles. At 23°C, all recombinant subunits appeared as oligomers of fast relaxation times, with calculated *R_h_* of ∼4 nm. Upon temperature increase, rAgB8/2 and rAgB8/3 formed other oligomeric states, detected by slower relaxation modes. As also observed in native PAGE, rAgB8/3 was the only subunit able to form higher-order oligomers, which are represented by the slow relaxation mode of ∼6 µs. The different oligomerization properties of AgB subunits in solution was also confirmed by SLS data (data not shown), with rAgB8/3 subunit being more prone to oligomerization.

We do not know whether the oligomeric states detected by DLS exactly correspond to those observed in native PAGE, but the differential oligomerization behavior of AgB recombinant subunits was detected by both techniques.

### Ultrastructural characterization of AgB oligomers

**Figure 4:**
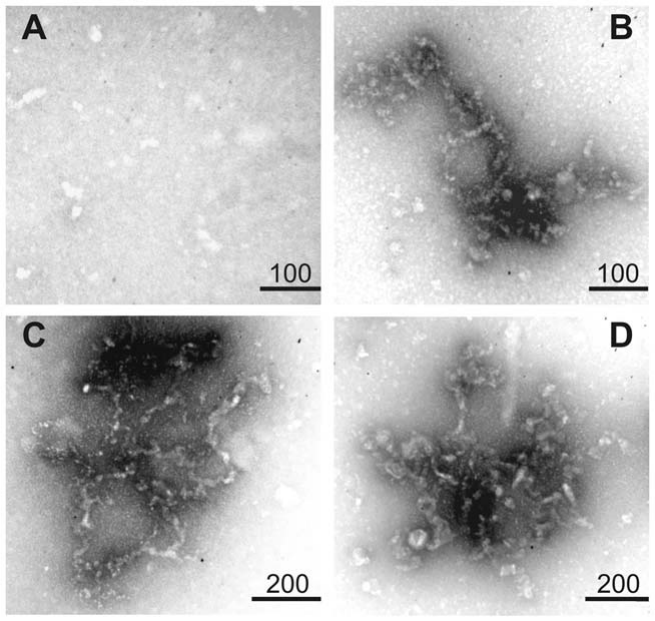
Ultrastructure of the AgB oligomers analyzed by TEM. TEM images from rAgB8/1 (A), rAgB8/2 (B), rAgB8/3 (C), and AgB (D) oligomers. Recombinant AgB subunits were incubated at 37°C before sample preparation for TEM analysis. (Scale bar in nm).

**Figure 5:**
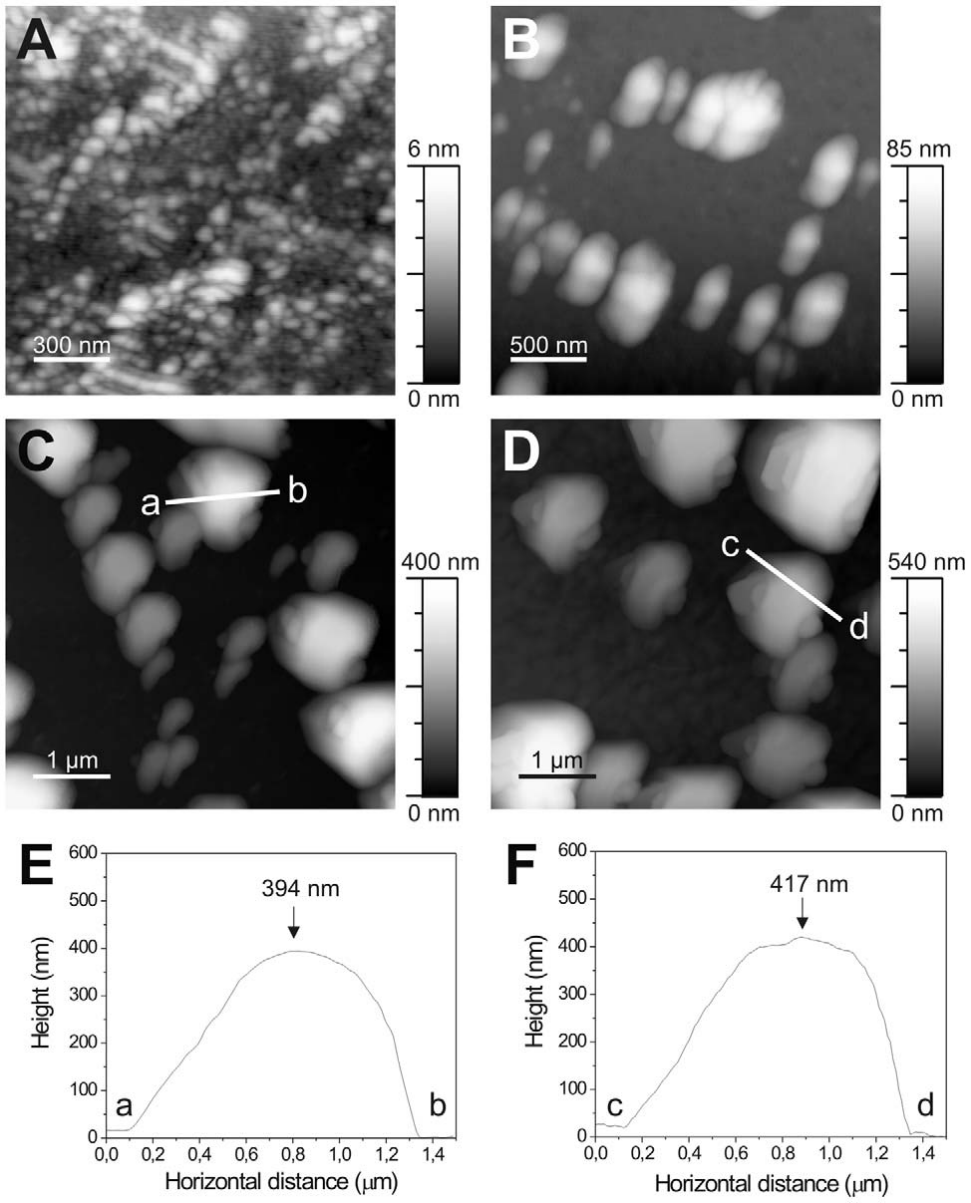
AFM 3D surface morphology of AgB oligomers. AFM height images for the rAgB8/1 (A), rAgB8/2 (B), rAgB8/3 (C), and AgB (D) oligomers. AgB subunits were incubated at 37°C for 15 min before sample preparation for AFM analysis. (E and F) AFM surface profiles along the a–b and c–d axes in C and D, respectively.

Image techniques (TEM and AFM) were used in order to characterize the ultrastructure of AgB oligomers and its recombinant counterparts formed at 37°C. The differential oligomerization behavior of AgB recombinant subunits were also detected by microscopy experiments (Figures 4 and 5), with a rAgB8/3>rAgB8/2>rAgB8/1 oligomer size relation. In the three-dimensional AFM images, the AgB oligomers display a near-globular shape and showed heterogeneity in size and morphology both between and within samples (Figure 5A–D). Among recombinant subunits, the oligomeric states formed by rAgB8/3 subunit were more similar, both in size and morphology, to those observed for *E. granulosus* AgB (Figure 5C–F).

### Structural stability of AgB recombinant oligomers

**Figure 6:**
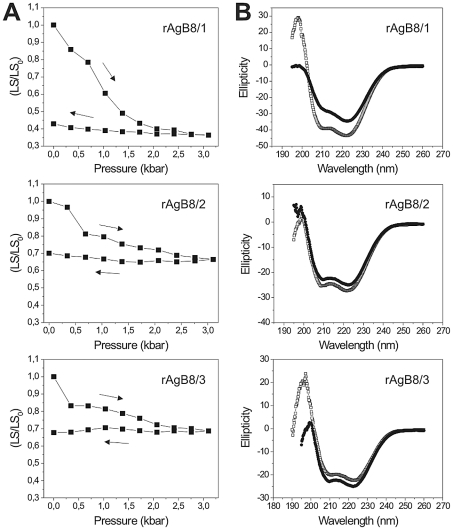
Pressure-induced dissociation of AgB recombinant oligomers and its effects on protein secondary structure. High hydrostatic pressure-treatment (A) and circular dichroism spectra (B) of the recombinant AgB oligomers. Protein samples (0.2 mg/ml in 25 mM tris buffer pH 7.5) were submitted to compression (→) and decompression (←) at 25°C, and light scattering was monitored at the steady state [LS was recorded and divided by the initial value (LS/LS_0_)]. Circular dichroism spectra were recorded before (open symbols) and immediately after (solid symbols) pressure treatment.

To compare the structural stabilities of AgB recombinant oligomers, proteins were subjected to treatment with high hydrostatic pressures, during which their oligomeric states were monitored by LS changes (Figure 6A). The LS signal of rAgB8/1 oligomers decreased by ∼60% after compression, which indicates its partial dissociation into smaller species. The rAgB8/2 and rAgB8/3 oligomers dissociated more poorly (LS decreased only ∼30%), even at the highest pressure attained in our experimental setup. The CD spectra recorded before and immediately after the compression of the samples (Figure 6B) indicated that the secondary structure for rAgB8/2 and rAgB8/3 was less perturbed by the pressure treatment than for rAgB8/1. Thus, pressure-induced dissociation experiments showed marked differences in the stabilities of oligomers formed by AgB subunits, with those formed by rAgB8/2 and rAgB8/3 subunits presenting greater structural stability. However, despite of the differences in the stability of recombinant AgB oligomers, the dissociation appears to be irreversible for all of them, since the LS values were maintained after pressure removal (Figure 6A).

**Figure 7:**
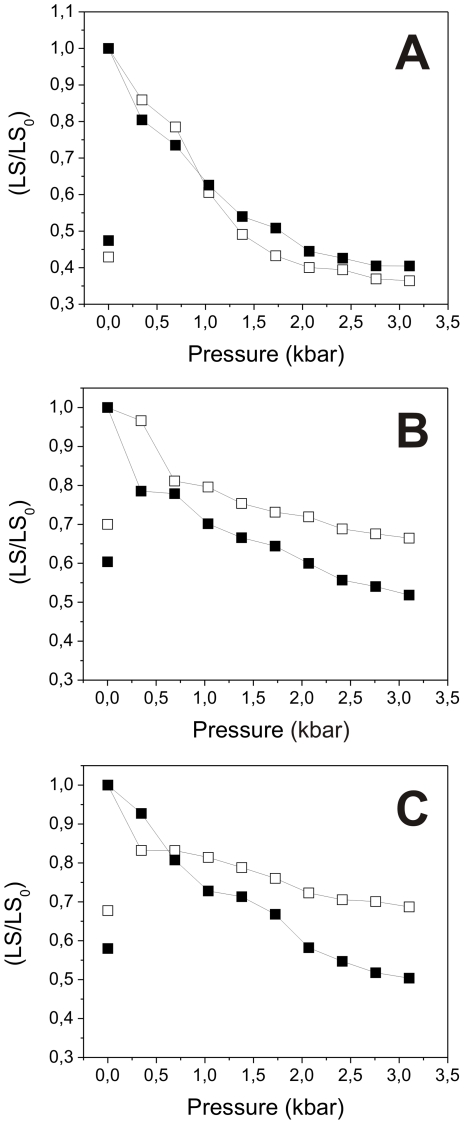
Effect of reducing agent on the pressure-induced dissociation of AgB recombinant oligomers. Effect of the 2-mercaptoethanol on the pressure-induced dissociation pattern of rAgB8/1 (A), rAgB8/2 (B), and rAgB8/3 (C) oligomers. Protein samples (0.2 mg/ml in 25 mM Tris buffer pH 7.5) in either the absence (open symbols) or presence (solid symbols) of 2-mercaptoethanol were submitted to pressure treatment at 25°C, and light scattering was monitored at the steady state [LS was recorded and divided by the initial value (LS/LS_0_)]. Symbols on the left indicate the (LS/LS_0_) values after decompression.

As cysteine residues could contribute to the higher stability of the oligomers formed by rAgB8/2 and rAgB8/3 subunits, we also evaluated the effect of the reducing agent 2-mercaptoethanol on the stability of the AgB recombinant oligomers (Figure 7). The reducing agent had no effect on rAgB8/1 oligomer dissociation (Figure 7A), while the rAgB8/2 and rAgB8/3 oligomers exhibited greater dissociation in the presence of the reducing agent (Figure 7B and C, respectively). Even in the presence of 2-mercaptoethanol, the rAgB8/2 and rAgB8/3 oligomers were somewhat more resistant to pressure-induced disassembly than those formed by the rAgB8/1 subunit. Nevertheless, the results indicate that the differential stability of recombinant oligomers primarily results from disulfide bond formation.

## Discussion

Few studies on *Echinococcus* AgB have addressed expression of the different AgB subunits in the hydatid cyst [22], [23] or identified the 8-kDa subunits involved in the formation of the AgB oligomers secreted in the hydatid fluid [17], [20]. Moreover, these previous studies analyzed a mixture of hydatid fluids from different cysts. In this work, we provided the first view of subunit composition for AgB samples purified from individual *E. granulosus* hydatid cysts from different hosts. Different experimental strategies, such as mass spectrometry and PAGE, allowed us to identify the subunits present in distinct oligomeric states of AgB. We also provided information on the relative abundance of AgB subunits using emPAI, a index that has been successfully employed to estimate relative abundance and stoichiometry of subunits on multiprotein complexes [31], [32].

AgB8/1, AgB8/2, AgB8/3 and AgB8/4 subunits were identified in all samples analyzed, with some quantitative and qualitative variations in AgB subunit composition within and between samples. The expression of different AgB subunits and their genomic variants may reflect an adaptive response of the parasite to different hosts and physiological conditions, diversifying AgB biochemical functions and antigenicity to promote its survival and evade host immune response [21], [33].

The *AgB* genes are highly polymorphic and several members are reported for each *AgB* subfamily [15], [21], [33]–[35]. This variation was detected here at protein level by the identification of an AgB8/2 variant, AgB8/2v8, which was previously genetically characterized in *E. granulosus* G1 strain [34]. The *AgB2* subfamily was previously reported as subjected to selective pressure, which might be result of the direct interaction between AgB and host immune system [21], [34]. This can be addressed in future studies of the effect of different AgB8/2 epitopes in the modulation of host responses.

Our results on AgB subunit composition are in agreement with the transcriptional data available for *AgB* genes, which show that *AgB1*, *AgB2*, *AgB3* and *AgB4* genes are expressed in *Echinococcus* metacestodes [22], [23]. We were not able to identify the AgB8/5 subunit in any of the samples studied, which suggests its absence or underrepresentation in the hydatid fluid. In agreement with our findings, *AgB5* transcripts were detected at very low levels in the larval stage of *E. granulosus* and *E. multilocularis*, increasing its expression in the adult worm [22], [23], which suggests adult-specific functions for AgB8/5. An assumed AgB8/5 subunit was recently reported in a proteomic analysis of *E. granulosus* hydatid fluid [20], but the identified sequence, although initially named AgB8/5 [21], was latter assigned to the *AgB3* subfamily [23], [36] and, therefore, does not correspond to the AgB8/5 subunit described by Mamuti et al. [16].

The detected AgB subunit relative abundance is also in agreement with the transcriptional data, which show that *AgB1*, *AgB2* and *AgB4* are transcribed at higher levels by the *E. granulosus* larval stage, whereas are poorly transcribed in protoscolex and other parasite developmental stages [23]. Therefore, both protein and RNA data suggest specific functions for AgB8/1, AgB8/2 and AgB8/4 subunits in AgB host-parasite relationships during metacestode chronic infection. Although it is not known which parasite tissue contributes more to the synthesis of AgB secreted in the hydatid fluid, our results show that the repertoire of secreted subunits resemble the expression profile of the germinal layer, suggesting that AgB secreted in the hydatid fluid is mostly produced by germinal layer cells.

*AgB3* gene, on the other hand, is expressed in all parasite stages and its level increases during development towards the adult worm [22], [23], which might indicate a more specific role for AgB8/3 subunit in adult worm differentiation. The observed discrepancies in AgB8/3 abundance between bovine and human cysts suggest that *AgB* genes, besides being developmentally regulated, may also vary their expression between parasite cysts and/or hosts. Comparing protein and RNA data from different cysts and hosts, one can note that the transcriptional profile of *AgB* genes described by Zhang et al. [23] for the germinal layer of a sheep cyst is more similar to the subunit abundance profile detected here for the human cyst than those observed for bovine cysts.

Altogether, the issues raised here point to the need of comparative studies using a larger number of single-cyst AgB samples from parasites of different hosts, strains and physiological states (fertile and infertile) to produce a clearer and more comprehensive view on the AgB subunit expression at RNA and protein levels. In this sense, the present work represents a first step in this direction, providing and standardizing methodologies for preparation and analysis of AgB samples from single *E. granulosus* cysts.

AgB subunit abundance can be correlated to some immunological features of 8-kDa subunits. The high antigenicity reported for AgB8/1 in *Echinococcus* infections [10], [37]–[39] could be related to its high expression in the parasite metacestode, as detected here at the protein level, and previously shown in transcriptional studies [22], [23]. The AgB8/2 subunit has been also described with a high diagnostic performance for CHD [9], [40] and was found here well represented in bovine and human cysts. The AgB8/4 subunit was also detected as relatively abundant in our cyst samples, but it has not been object of immunological characterization so far. In addition, AgB8/2 and AgB8/4 present 70% homology in their amino acids sequences [15] and are likely to share some antigenic epitopes, reinforcing the need to test AgB8/4 immunodiagnostic potential. On the other hand, AgB8/3 subunit showed lower antigenicity in preliminary tests [our unpublished results] and discrepant abundances between cyst samples. Further studies are needed to test possible implications of structural aspects of AgB oligomer architecture on subunit antigenicity, such as epitope masking or limited processing and presentation to T cells [18], [19]. In addition, immunoassays using combinations of AgB subunits, or mixtures of synthetic peptides containing major epitopes from different subunits, may improve the performance of serodiagnosis for echinococcosis [10], [41].

Since its first description by Oriol et al. [2], the oligomeric states of AgB have received little attention. Here, using AgB purified from hydatid fluid and recombinant 8-kDa subunits we characterized the *E. granulosus* AgB oligomers and demonstrated that they are not artifacts of the protein purification process and can be reproduced *in vitro*, under physiological conditions, using recombinant subunits. The results obtained in the present work for AgB structural analysis, besides confirming some previous findings [2], [18], have extended the structural characterization of recombinant oligomers and generated a useful comparison to those formed by *E. granulosus* AgB.

The structural analysis of recombinant subunits also provided some insights into AgB oligomer formation and stability. Subunits found to be involved in the formation of AgB high-order oligomers were detected as having different oligomerization properties. Thus, considering subunit hetero-oligomerization, we could hypothesize a scenario where the more aggregative subunits act as nucleation centers for the formation of higher-order oligomers, to which less aggregative subunits could be attached. We also cannot exclude the possibility that post-translational modifications, absent in the recombinant proteins, could alter the oligomerization properties detected here for each 8-kDa subunit. AgB post-translational modifications, as previously described lipidation [2], may also be involved in the covalent association between subunits and in the oligomeric structure maintenance, since recombinant oligomers are non-covalently associated and completely dissociate under SDS-PAGE reducing conditions. Our structural analysis also points that AgB8/2 and AgB8/3 subunits could contribute to a higher stability of *E. granulosus* AgB oligomers, as they showed greater structural stability, which primarily results from disulfide bond formation.

Other issue that remains elusive and deserves future investigation is the site of AgB subunit oligomerization. Secretory proteins are synthesized in the endoplasmic reticulum and oligomerization typically occurs within this cellular compartment, although, in some cases, oligomerization takes place in the intermediate compartment and Golgi apparatus [42]. Large protein oligomers, as those observed for *E. granulosus* AgB, were reported to be secreted by both lower and higher eukaryotes [43]–[45], with protein oligomerization occurring within the cisternae of Golgi apparatus. However, we do not know so far whether AgB higher-order oligomers can be formed within the secretory pathway of the germinal cells or they are formed only after secretion, in the hydatid fluid.

Overall, this work provided important information on the AgB subunit composition, abundance and oligomerization, significantly increasing the current knowledge on AgB protein expression and structure. Using recombinant subunits we were able to structurally characterize the oligomers formed by different AgB subunits and to compare them to those formed by *E. granulosus* AgB. Although further studies will be required to completely elucidate AgB structure, our results will help understanding AgB roles in the host-parasite interplay during *Echinococcus* infection [3], [46], [47].

## Supporting Information

Table S1 Subunits identified by mass spectrometry analysis of AgB from bovine and human hydatid cysts.
(DOC)

Text S1 Supporting methods.
(DOC)
