# Supplementary material for: Echinococcus granulosus Antigen B Structure: Subunit Composition and Oligomeric States
Source: PLoS Negl Trop Dis. 2012 Mar 6;6(3):e1551. doi: 10.1371/journal.pntd.0001551 (PMC3295803; doi:10.1371/journal.pntd.0001551)
Supplement: Table S1 — Subunits identified by mass spectrometry analysis of AgB from bovine and human hydatid cysts. (DOC) [file pntd.0001551.s001.doc]

**Table S1.** Subunits identified by mass spectrometry analysis of AgB from bovine and human hydatid cysts.

| Cyst | Sample | AgB subunit | MASCOT score | Cov (%) | Peptide matches | emPAI |
| --- | --- | --- | --- | --- | --- | --- |
| Bovine (cyst 1) | in-solution digested | | | | | |
|  |  | AgB8/1 | 414 | 43 | 14 | 12.80 |
|  |  | AgB8/2 / AgB8/2v8 | 105 | 20 | 2 | 0.50 |
|  |  | AgB8/3 | 55 | 14 | 2 | 0.52 |
|  |  | AgB8/4 | 288 | 44 | 4 | 1.23 |
|  | in-gel digested (SDS-PAGE) | | | | | |
|  | Monomer | AgB8/1 | 254 | 55 | 12 | 5.83 |
|  |  | AgB8/2v8 | 267 | 52 | 8 | 3.23 |
|  |  | AgB8/4 | 110 | 35 | 2 | 0.42 |
|  | Dimer | AgB8/1 | 258 | 55 | 16 | 9.02 |
|  |  | AgB8/2v8 | 188 | 44 | 4 | 1.06 |
|  |  | AgB8/3 | 41 | 14 | 2 | 0.45 |
|  |  | AgB8/4 | 138 | 45 | 4 | 1.02 |
|  | Trimer | AgB8/1 | 222 | 55 | 8 | 3.65 |
|  |  | AgB8/2v8 | 207 | 44 | 4 | 1.06 |
|  |  | AgB8/3 | 22 | 14 | 1 | 0.45 |
|  |  | AgB8/4 | 207 | 40 | 6 | 1.02 |
|  | Tetramer | AgB8/1 | 58 | 32 | 2 | - |
|  |  | AgB8/2v8 | 25 | 34 | 2 | - |
|  |  | AgB8/4 | 71 | 28 | 2 | - |
|  | Pentamer | AgB8/1 | 20 | 32 | 1 | - |
|  |  | AgB8/2 / AgB8/2v8 | 23 | 20 | 1 | - |
|  |  | AgB8/4 | 54 | 28 | 2 | - |
|  | Hexamer | AgB8/1 | 42 | 15 | 1 | - |
|  |  | AgB8/2 / AgB8/2v8 | 18 | 20 | 1 | - |
|  | Heptamer | AgB8/2 / AgB8/2v8 | 19 | 20 | 1 | - |
|  | in-gel digested (native PAGE) | | | | | |
|  | ~550 kDa band | AgB8/1 | 264 | 40 | 12 | 5.83 |
|  |  | AgB8/2v8 | 194 | 46 | 9 | 1.95 |
|  |  | AgB8/3 | 54 | 14 | 1 | 0.45 |
|  |  | AgB8/4 | 251 | 55 | 9 | 3.09 |
|  | Smear | AgB8/1 | 407 | 40 | 16 | 9.02 |
|  |  | AgB8/2 | 399 | 57 | 11 | 3.16 |
|  |  | AgB8/2v8 | 367 | 56 | 12 | 3.23 |
|  |  | AgB8/3 | 53 | 14 | 2 | 0.45 |
|  |  | AgB8/4 | 401 | 47 | 14 | 4.81 |
| Bovine (cyst 2) | in-solution digested | | | | | |
|  |  | AgB8/1 | 1118 | 61 | 34 | 7.91 |
|  |  | AgB8/2 | 140 | 20 | 3 | 0.50 |
|  |  | AgB8/3 | 42 | 14 | 2 | 0.52 |
|  |  | AgB8/4 | 268 | 44 | 7 | 3.96 |
|  | in-gel digested (SDS-PAGE) | | | | | |
|  | Monomer | AgB8/1 | 148 | 32 | 6 | 2.17 |
|  |  | AgB8/2 | 40 | 20 | 1 | 0.43 |
|  |  | AgB8/3 | 22 | 14 | 1 | 0.45 |
|  |  | AgB8/4 | 38 | 28 | 2 | 0.42 |
|  | Dimer | AgB8/1 | 100 | 32 | 6 | 2.17 |
|  |  | AgB8/2 | 45 | 20 | 2 | 0.43 |
|  |  | AgB8/3 | 22 | 14 | 1 | 0.45 |
|  |  | AgB8/4 | 178 | 45 | 4 | 1.02 |
|  | Trimer | AgB8/1 | 101 | 24 | 2 | 0.47 |
|  |  | AgB8/2 | 33 | 35 | 2 | 0.43 |
|  |  | AgB8/4 | 268 | 45 | 10 | 4.81 |
|  | Tetramer | AgB8/2 | 18 | 20 | 1 | - |
|  |  | AgB8/4 | 60 | 28 | 2 | - |
|  | Pentamer | AgB8/1 | 52 | 24 | 1 | - |
|  |  | AgB8/2 | 16 | 20 | 1 | - |
|  |  | AgB8/4 | 67 | 32 | 2 | - |
|  | in-gel digested (native PAGE) | | | | | |
|  | ~550 kDa band | AgB8/1 | 359 | 26 | 8 | 2.17 |
|  |  | AgB8/2 | 373 | 45 | 10 | 1.91 |
|  |  | AgB8/3 | 43 | 14 | 2 | 0.45 |
|  |  | AgB8/4 | 550 | 44 | 12 | 3.09 |
|  | Smear | AgB8/1 | 164 | 16 | 6 | 1.16 |
|  |  | AgB8/2 | 356 | 35 | 8 | 1.04 |
|  |  | AgB8/3 | 52 | 14 | 1 | 0.45 |
|  |  | AgB8/4 | 513 | 70 | 20 | 7.26 |
| Human | in-solution digested | | | | | |
|  |  | AgB8/1 | 1443 | 52 | 41 | 30.74 |
|  |  | AgB8/2 | 157 | 44 | 5 | 7.47 |
|  |  | AgB8/3 | 406 | 25 | 12 | 8.19 |
|  |  | AgB8/4 | 723 | 45 | 18 | 4.81 |
|  | in-gel digested (SDS-PAGE) | | | | | |
|  | Monomer | AgB8/1 | 562 | 46 | 21 | 4.75 |
|  |  | AgB8/2 | 23 | 10 | 1 | 0.50 |
|  |  | AgB8/3 | 65 | 14 | 1 | 0.52 |
|  | Dimer | AgB8/1 | 271 | 46 | 8 | 4.75 |
|  |  | AgB8/2 | 82 | 18 | 10 | 1.25 |
|  |  | AgB8/3 | 72 | 14 | 2 | 0.52 |
|  |  | AgB8/4 | 80 | 37 | 4 | 1.25 |
|  | Trimer | AgB8/1 | 41 | 13 | 1 | 0.55 |
|  |  | AgB8/2 | 91 | 21 | 6 | 2.37 |
|  |  | AgB8/4 | 45 | 10 | 4 | 0.50 |
|  | in-gel digested (native PAGE) | | | | | |
|  | ~550 kDa | AgB8/1 | 627 | 40 | 17 | 9.02 |
|  |  | AgB8/2 | 531 | 57 | 13 | 4.93 |
|  |  | AgB8/3 | 91 | 29 | 2 | 1.09 |
|  |  | AgB8/4 | 383 | 55 | 12 | 7.26 |
|  | Smear | AgB8/1 | 527 | 40 | 11 | 6.58 |
|  |  | AgB8/2 | 367 | 47 | 8 | 1.56 |
|  |  | AgB8/3 | 54 | 29 | 2 | 0.92 |
|  |  | AgB8/4 | 491 | 65 | 21 | 5.41 |

Cov: sequence coverage.
