# Supplementary material for: Echinococcus granulosus Antigen B Structure: Subunit Composition and Oligomeric States
Source: PLoS Negl Trop Dis. 2012 Mar 6;6(3):e1551. doi: 10.1371/journal.pntd.0001551 (PMC3295803; doi:10.1371/journal.pntd.0001551)
Supplement: Text S1 — Supporting methods. (DOC) [file pntd.0001551.s002.doc]

**Genotyping of parasite material**

Genotyping of bovine and human cysts was carried out with hydatid fluid particulate material. One milliliter of each bovine and human hydatid fluid was centrifuged at 20000 x g for 15 min at 4°C and the resulting pellet was resuspended in 10 µl of MilliQ water. The resuspended material was incubated at 100°C for 5 min and clarified by a brief centrifugation. One or five microliters of each processed sample were used as DNA template in a PCR reaction for amplification of a 391-bp fragment of the mitochondrial cytochrome C oxidase 1 (CO1) gene [1]. PCR products were purified using a GFX column (GE Healthcare, Chalfont St. Giles, UK) and sequenced using a MegaBace 1000 DNA Analysis System (GE Healthcare).

**Analysis of DLS data**

The time-dependent fluctuations in the scattered intensity, which resulted from the random thermal motion of the particles, were analyzed using a digital correlator and processed as an autocorrelation function [2]. Normalized electric field autocorrelation functions *g*_1_(*t*), which were calculated from *g*_2_(*t*), were analyzed using GENDIST software, which employs the REPES algorithm [3] to perform the inverse Laplace transformation as demonstrated in Eq. 1:

$g_{2}\left(t\right)-1=\beta\left[\int A\left(\tau\right)\exp\left(-\frac{t}{\tau}\right)\text{d}\tau\right]^{2}$ (1)

where *t* is the delay time of the correlation function and *β* is an instrumental parameter known as contrast. The resulting *A(τ)* is a distribution of relaxation times that generally consists of several peaks representing individual dynamic processes. Herein, the distributions of the relaxation times are demonstrated in the equal area representation [4] as *τA*(*τ*) vs. log *τ*.

Each relaxation time *τ* and relaxation frequency *Γ* $\left(\tau^{-1}\right)$ that is characteristic of a dynamic process can be quantitatively associated with an apparent diffusion coefficient (*D*), determined according to the following relation:

$D=\frac{\Gamma}{q^{2}}$ (2)

Finally, the apparent hydrodynamic radius *R_h_* is derived from the diffusion coefficient (*D*) using the well-known Stokes-Einstein relation:

$R_{h}=\frac{k_{B}T}{6\pi\eta D}$ (3)

where *k*_B_ is the Boltzmann constant, *T* is the absolute temperature, and *η* is the viscosity of the solvent at the same temperature.

**Analysis of SLS data**

The scattered light intensities were plotted as Rayleigh ratio using the following equation:

$R_{\text{S}}=\left(\frac{I_{\text{S}}-I_{\text{PBS}}}{I_{\text{tol}}}\right)\times R_{\text{tol}}$ (4)

where *R*_s_ is the Rayleigh ratio of the protein solution; *I*_s_, *I*_PBS_ and *I*_tol_ are the average light scattering intensities of the solution, buffer and toluene, respectively; and *R*_tol_ is the Rayleigh ratio of toluene.

**References**

1. Bowles J, Blair D, McManus DP (1992) Genetic variants within the genus *Echinococcus* identified by mitochondrial DNA sequencing. Mol Biochem Parasitol 54: 165-173.

2. Berne BJ, Pecora R (1976) Dynamic Light Scattering. New York: John Wiley. 376 p.

3. Jakeš J (1995) Regularized Positive Exponential Sum (REPES) Program - A way of inverting Laplace transform data obtained by dynamic light scattering. Collect Czechos Chem Communic 60: 1781-1797.

4. Štěpánek P (1993) Data analysis in dynamic light scattering. In: Brown W, editor. Dynamic Light Scattering: The Method and Some Applications. Oxford: Oxford Science Publications. pp. 177-241.
